# A Methodological Quality Assessment of Meta‐Analyses on Sleep Disorder Treatments Using AMSTAR 2

**DOI:** 10.1002/brb3.70140

**Published:** 2024-11-17

**Authors:** Leonard Ho, Yan Ling Kwok, Xi Chen, Irene X. Y. Wu, Chen Mao, Vincent Chi Ho Chung

**Affiliations:** ^1^ Jockey Club School of Public Health and Primary Care, Faculty of Medicine The Chinese University of Hong Kong Shatin New Territories Hong Kong; ^2^ Xiangya School of Public Health Central South University Changsha Hunan China; ^3^ Hunan Provincial Key Laboratory of Clinical Epidemiology Xiangya School of Public Health Changsha Hunan China; ^4^ Department of Epidemiology School of Public Health Southern Medical University Guangzhou Guangdong China; ^5^ School of Chinese Medicine Faculty of Medicine The Chinese University of Hong Kong Shatin New Territories Hong Kong

**Keywords:** evidence‐based practice, meta‐analysis, research design, sleep, systematic reviews

## Abstract

**Background**: Meta‐analyses (MAs) provide up‐to‐date, quantified evidence on treatment effects, which may be useful for clinical and policy decision‐making. However, the quality of MAs varies, and methodological flaws can limit their reliability.

**Aims**: This review evaluated the methodological quality of MAs on sleep disorder treatments.

**Methods**: We searched MEDLINE, EMBASE, and PsycINFO for eligible MAs on randomized controlled trials of sleep disorder treatments published between 2018 and 2023. We extracted MAs' bibliographical characteristics with a predesigned form and appraised their methodological quality using AMSTAR (A Measurement Tool to Assess Systematic Reviews) 2. We explored the associations between bibliographical characteristics and methodological quality ratings using Kruskal–Wallis tests and Spearman's rank correlation coefficients.

**Results/Outcomes**: Among the 104 MAs, the majority (*n* = 82; 78.9%) had critically low quality, 19 (18.3%) had low quality, and only 3 (2.9%) had high quality. Regarding AMSTAR 2 critical domains, 97 (93.3%) MAs did not provide a list of excluded studies and justify the exclusions, 75 (72.1%) did not use a comprehensive literature search strategy, and 56 (53.9%) lacked a registered protocol and did not justify protocol deviations. Cochrane reviews (*p* = 0.018), MAs with European corresponding authors (*p* < 0.001), and MAs receiving European funding (*p* < 0.001) performed better than their counterparts.

**Conclusions/Interpretation**: The methodological quality of recent MAs on sleep disorder treatments is unsatisfactory. Future reviewers should address the identified critical methodological issues. In addition, substantial resources and funding should be allocated to support training in evidence synthesis and critical appraisal for researchers and clinicians.

## Graphical abstract

The methodological quality of recent meta‐analyses on sleep disorder treatments is unsatisfactory, with the majority rated as critically low. Greater attention should be paid to providing a list of excluded studies with justifications for exclusions, using a comprehensive literature search strategy, registering a protocol prior to conducting reviews, and justifying any protocol deviations.

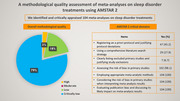

## 1 Introduction

Sleep disorders involve problems with the quality, timing, and amount of sleep, resulting in daytime distress and functional impairment (American Psychiatric Association 2023). They can be classified into seven categories: insomnia, sleep‐related breathing disorders, central disorders of hypersomnolence, circadian rhythm sleep–wake disorders, parasomnias, sleep‐related movement disorders, and other sleep disorders (Sateia 2014). Sleep disorders are prevalent, with estimates suggesting that up to 50% of the global population suffers from these conditions, depending on the definition used (National Institute for Health and Care Excellence 2022). Sleep disorders not only cause mental and physical health problems, such as depression and cardiovascular diseases (Buysse 2013), but also place a tremendous financial burden on healthcare systems and patients (Lee 2018). In the United States, the additional healthcare expenses and self‐expenditures for prescriptions associated with sleep disorders were estimated to be 6975 USD and 195 USD, respectively, in 2018 alone (Huyett and Bhattacharyya 2021).

Considering the broad spectrum of sleep disorders and their impact, resources have been devoted to developing various pharmacological and non‐pharmacological interventions aimed at improving patients' quality of life. For example, behavioral interventions and medications are commonly offered to patients with insomnia and central disorders of hypersomnolence (Schutte‐Rodin et al. 2008; Lopez et al. 2017). Similarly, there are continuous positive airway pressure treatments and medications, such as topical nasal corticosteroids, for obstructive sleep apnea (Sateia 2014). Clinicians, having to choose from a wide variety of options, must have access to reliable and up‐to‐date evidence to guide their selection of suitable interventions.

In evidence‐based practice, systematic review and meta‐analysis (MA) are considered the highest level of evidence supporting the effectiveness of interventions. Systematic reviews aim to synthesize and appraise the empirical findings that meet pre‐specified eligibility criteria, while MAs take a step further by quantitatively pooling effect estimates reported by the included primary studies in systematic reviews, if appropriate (The Cochrane Collaboration 2020). If conducted robustly, they are supposed to produce reliable evidence with minimal bias to inform clinical and policy decision‐making (Murad et al. 2016). Despite the rapid increase in the number of MAs in recent years, many of them are deemed unnecessary, unrigorously performed, and often produce conflicted results (Ioannidis 2016). If these publications are methodologically flawed, they may overestimate or underestimate treatment effects, which, in turn, mislead clinical and policy decision‐making (Mulrow, Cook, and Davidoff 1997). Therefore, evidence users should endeavor to critically appraise the methodological quality of MAs using validated tools to ensure their trustworthiness before adopting their conclusions (Murad et al. 2016).

Until now, the methodological quality of recent MAs on treatments for sleep disorders has not been systematically evaluated. In addition, it is not known whether particular bibliographic characteristics are linked to the overall methodological quality of MAs on this topic. For instance, a recent assessment of MAs on atopic dermatitis found that Cochrane reviews, updates of previous MAs, MAs with European corresponding authors, and MAs funded by European institutions tend to have better overall methodological quality, as do those with higher impact factors and more authors (Ho et al. 2024). These findings could provide insights into how to improve the methodological quality of future MAs and help evidence users increase their chances of identifying relatively high‐quality MAs within time limitations. Therefore, this review aims to (i) describe the bibliographic characteristics of a representative sample of MAs on sleep disorder treatments, (ii) appraise the methodological quality of these MAs using AMSTAR (A MeaSurement Tool to Assess systematic Reviews) 2, and (iii) investigate the associations between bibliographic characteristics and methodological quality.

## 2 Methods

### 2.1 Eligibility Criteria

We included MAs published in English within the previous 5 years (from January 2018 to March 2023) that focused on randomized controlled trials evaluating treatments for any sleep disorders documented in the International Classification of Sleep Disorders (third edition) (Sateia 2014). No restrictions were placed on the age groups or comorbidities of study populations. We excluded MAs on the prevention of sleep disorders or the treatment of relevant complications. Narrative reviews, protocols, network MAs, and clinical guidelines were also excluded.

### 2.2 Literature Search and Selection

We conducted a comprehensive literature search in MEDLINE, EMBASE, and PsycINFO on the Ovid platform to obtain representative samples of MAs. We applied filters for randomized controlled trials and MAs when searching for results to balance specificity and sensitivity (McMaster Health Information Research Unit 2023a, 2023b, 2023c). We restricted the scope of the literature search from January 2018 to March 2023 to identify the most up‐to‐date treatment strategies. Details of the search strategies are presented in Table S1.

We imported all retrieved citations into EndNote 20 (Clarivate, London, UK) for deduplication and management. Two reviewers (Y.L.K. and X.C.) independently screened the titles and abstracts and then assessed the full texts of potential MAs against the eligibility criteria. Any disagreements were resolved by a senior reviewer (L.H.).

### 2.3 Data Extraction and Methodological Quality Assessment

We extracted the bibliographical characteristics of included MAs with a predesigned 18‐item data extraction form, which has been used in several MA appraisals recently published (Ho et al. 2024; Zhong et al. 2022; Ho et al. 2021; Tsoi et al. 2020; Cheung et al. 2022; Wu et al. 2020; Ho et al. 2023). The 18 items were demonstrated to have potential associations with MA methodological quality. Table S2 illustrates the data extraction form used in this study.

We used the critical appraisal tool, AMSTAR 2, to evaluate the methodological quality of included MAs (Shea et al. 2017). It has a total of 16 items corresponding to 16 domains important to methodological rigor. Eleven domain items (Items 1, 3, 5, 6, 10, 11, 12, 13, 14, 15, and 16) are rated with two options (yes/no), depending on whether the MA fulfills the criteria for standard methods and reporting. Five domain items (Items 2, 4, 7, 8, and 9) are rated with three options (yes/no/partially yes). Among the 16 domain items of AMSTAR 2, the following seven are considered to critically affect the validity of an MA and its conclusions, and therefore, are classified as “critical” domain items:

- Registering an a priori protocol to outline review methods and justifying protocol deviations (Item 2)
- Using a comprehensive literature search strategy (Item 4)
- Clearly listing excluded primary studies and justifying study exclusions (Item 7)
- Assessing the risk of bias in primary studies (Item 9)
- Employing appropriate MA methods (Item 11)
- Considering the risk of bias in primary studies when interpreting MA results (Item 13)
- Evaluating publication bias and discussing its likely impact on MA results (Item 15)

AMSTAR 2 provides guidance to reach a conclusion on an MA's overall methodological quality (i.e., high, moderate, low, or critically low) based on that MA's performance across the domains. Table S3 outlines all 16 domain items and the criteria for evaluating the overall methodological quality of an MA. Two reviewers (Y.L.K. and X.C.) independently performed data extraction and quality assessment. Conflicts were resolved by consensus or, if necessary, by a senior reviewer (L.H.).

### 2.4 Data Analysis

We summarized the bibliographic characteristics of included MAs and the AMSTAR 2 critical appraisal results using descriptive statistics, with frequencies and percentages for categorical variables and medians and ranges for continuous variables. Using Kruskal–Wallis tests, we investigated differences in the overall methodological quality of MAs across the categorical variables. The categorical variables included in the analysis were “being a Cochrane review” (yes/no), “being an updated MA” (yes/no), “reported intervention harms” (yes/no), “searched non‐English databases” (yes/no), “included a PRISMA‐like flow diagram” (yes/no), “location of corresponding author” (multiple options), “funding location” (multiple options), “type of interventions” (multiple options), “reported year of coverage of literature search” (yes/no), “search terms reported for one or more electronic databases” (multiple options), “language of included primary studies” (multiple options), and “tools for assessing quality of primary studies” (multiple options). Similarly, we explored the differences in MAs' overall methodological quality across the continuous variables, including “publication year,” “publication journal impact factor,” “number of review authors,” “number of included primary studies,” and “number of participants included in primary studies.” A *p* value of < 0.05 was considered statistically significant. We conducted these data analyses using SPSS Statistics version 26 (IBM Corporation, Armonk, New York, USA).

## 3 Results

### 3.1 Literature Search and Selection

**FIGURE 1:**
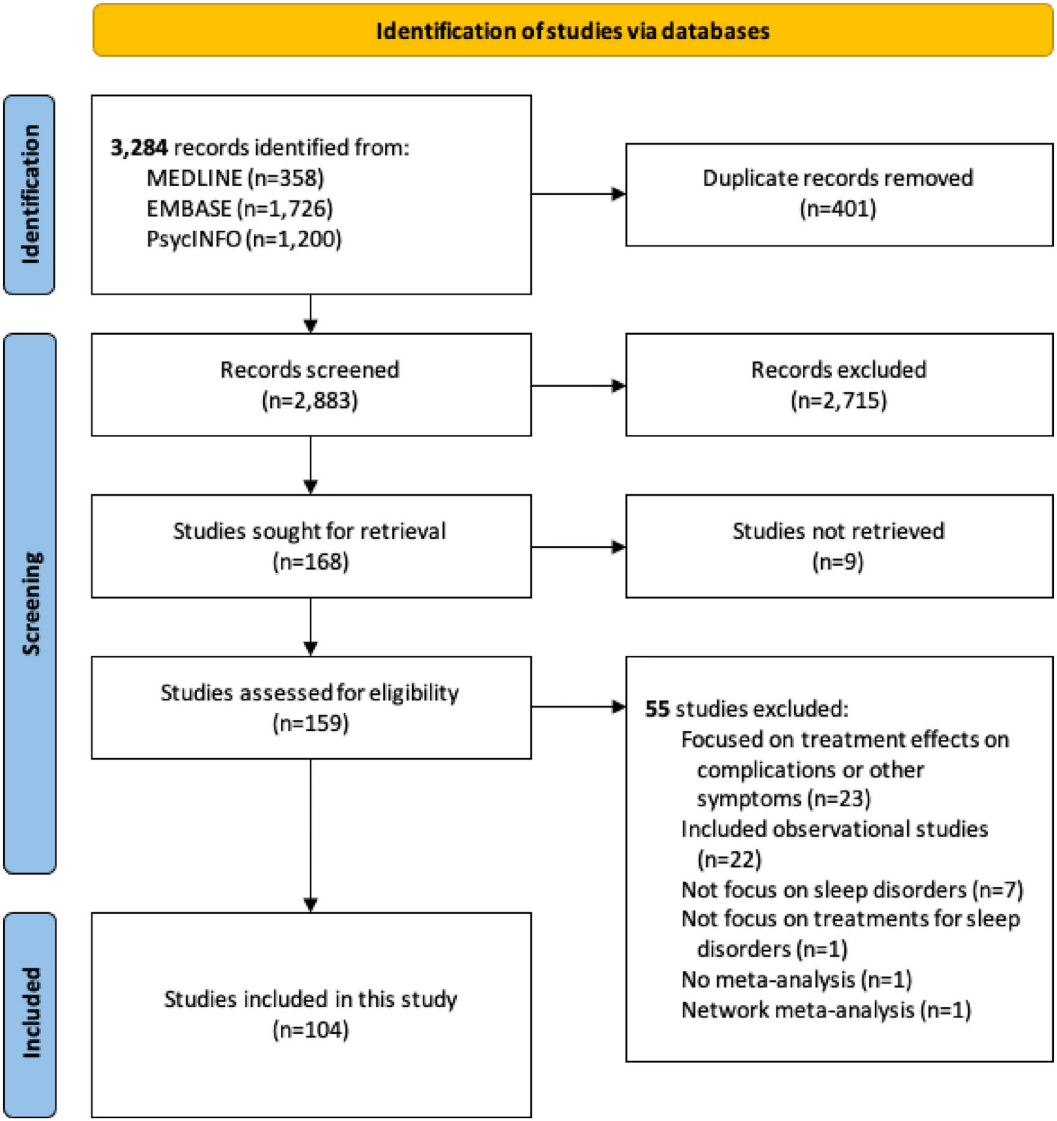
Literature search and selection process.

The literature search yielded 3284 records. After deduplication, we screened 2883 records by titles and abstracts and then assessed 159 records by full text. Finally, a total of 104 MAs fulfilled the eligibility criteria and were included in this study. Figure 1 visualizes the literature search and selection. Table S4 provides a list of excluded records with justifications.

### 3.2 Bibliographical Characteristics of MAs on Sleep Disorder Treatments

Table 1 summarizes the bibliographical characteristics of the included 104 MAs. The 104 MAs synthesized a total of 1855 primary studies, which included 185,224 participants with sleep disorders. Most of the MAs (99.0%) were non‐Cochrane reviews, and one (1.0%) was an update of a previous review. Over half (67 MAs; 64.4%) of the MAs focused only on non‐pharmacological interventions, and 31 (29.8%) focused only on pharmacological interventions. Forty‐two (40.4%) MAs reported the harms or adverse events of interventions. Over half of the corresponding authors and funding were from Asian countries (71 MAs; 68.3% for both characteristics), while none were from the African continent.

**TABLE 1:** Bibliographical characteristics of the 104 included meta‐analyses on sleep disorder treatments.

Bibliographical characteristics	Results^a^, ^b^
Cochrane review	1 (1.0)
An update of a previous meta‐analysis	1 (1.0)
Meta‐analyses reporting intervention harms	42 (40.4)
Meta‐analyses that searched English databases	104 (100)
Meta‐analyses that searched non‐English databases	53 (51.0)
Included a PRISMA‐like flow diagram	102 (98.1)
Publication year median (range)	2021 (2020–2023)
Publication journal impact factor median (range)	2.94 (0.0–15.36)
Number of review authors median (range)	6 (1–15)
Number of included primary studies	
Total	1855
Median across the meta‐analyses (range)	14 (3–124)
Number of participants included in primary studies	
Total	185224
Median across the meta‐analyses (range)	1176 (151–13227)
Location of corresponding author	
Europe	13 (12.5)
America	15 (14.4)
Asia	71 (68.3)
Oceania	5 (4.8)
Africa	0 (0)
Funding location of meta‐analysis	
Europe	3 (2.9)
America	13 (12.5)
Asia	71 (68.3)
Oceania	5 (4.8)
Africa	0 (0)
Not reported	12 (11.5)
Type of interventions	
Pharmacological	67 (64.4)
Non‐pharmacological	31 (29.8)
Both types	6 (5.8)
Reported year of coverage of literature search	103 (99.0)
Search terms reported for one or more electronic databases	
No research term	0 (0)
Topics/free text/keywords/MeSH	54 (51.9)
Full Boolean	50 (48.1)
Readers are referred elsewhere for full search strategy	0 (0)
Language of included primary studies in the meta‐analyses	
English only	12 (11.5)
Language other than English	1 (1.0)
English and languages other than English	40 (38.5)
Language criteria not reported	51 (49.0)
Tools for assessing quality of primary studies	
Cochrane risk of bias	72 (69.2)
Jadad Scale	5 (4.8)
More than one tools	2 (1.9)
Others	25 (24.0)

Abbreviations: MeSH, Medical Subject Headings; PRISMA, Preferred Reporting Items for Systematic Reviews and Meta‐Analysis.

^a^ Values are *N* (%), or median (range).

^b^ Percentages were calculated by using the total number of the categories as the denominator.

All MAs searched English databases, but only 53 (51.0%) of them searched non‐English databases. Around half (51 MAs; 49.0%) of them did not report whether language restrictions were applied to the literature search. Nearly all MAs had a PRISMA‐like flow diagram (102 MAs; 98.1%) and reported the year of coverage comprehensively (103 MAs; 99.0%). The most (72 MAs; 69.2%) commonly adopted methodological quality assessment tool was the Cochrane risk of bias tool.

### 3.3 Methodological Quality of MAs

#### 3.3.1 Performance Across the AMSTAR 2 Domains

Table 2 presents the results of the AMSTAR 2 assessment of the 104 included MAs. The included MAs generally performed well on 10 out of 16 domains, with more than 90% fulfilling the relevant criteria: (i) all included PICO components in their research questions and inclusion criteria (Item 1); (ii) all explained their selection of the study designs for inclusion (Item 3); (iii) 100 (96.2%) MAs described the included primary studies in adequate detail (Item 8); (iv) 102 (98.1%) MAs assessed the risk of bias in primary studies (Item 9); (v) all employed appropriate methods for the MA (Item 11); (vi) all assessed the potential impact of risk of bias in primary studies on the MA (Item 12); (vii) all took into account the risk of bias in primary studies when interpreting the MA results (Item 13); (viii) all explained and discussed the heterogeneity observed in the MA comprehensively (Item 14); (ix) all evaluated publication bias and discussed its likely impact on the MA results (Item 15); and (x) 103 (99.0%) MAs reported their potential sources of conflict of interest (Item 16). Four of the above domain items (Item 9, Item 11, Item 13, and Item 15) are linked to critical AMSTAR 2 domains.

**TABLE 2:** Results of the AMSTAR2 domain items for the 104 included meta‐analyses on sleep disorder treatments.

AMSTAR 2 items	Yes (%)	Partial yes (%)	No (%)
1. Did the research questions and inclusion criteria for the review include the components of PICO?	104 (100)	NA	0 (0)
2. Did the report of the review contain an explicit statement that the review methods were established prior to the conduct of the review and did the report justify any significant deviations from the protocol?^a^	47 (45.2)	1 (1.0)	56 (53.8)
3. Did the review authors explain their selection of the study designs for inclusion in the review?	104 (100)	NA	0 (0)
4. Did the review authors use a comprehensive literature search strategy?^a^	29 (27.9)	75 (72.1)	0 (0)
5. Did the review authors perform study selection in duplicate?	90 (86.5)	NA	14 (13.5)
6. Did the review authors perform data extraction in duplicate?	84 (80.8)	NA	20 (19.2)
7. Did the review authors provide a list of excluded studies and justify the exclusions?^a^	7 (6.7)	0 (0)	97 (93.3)
8. Did the review authors describe the included studies in adequate detail?	100 (96.2)	4 (3.8)	0 (0)
9. Did the review authors use a satisfactory technique for assessing the RoB in individual studies that were included in the review?^a^	102 (98.1)	0 (0)	2 (1.9)
10. Did the review authors report on the sources of funding for the studies included in the review?	75 (72.1)	NA	29 (27.9)
11. Did the review authors use appropriate methods for statistical combination of results?^a^	104 (100)	NA	0 (0)
12. Did the review authors assess the potential impact of RoB in individual studies on the results of the meta‐analysis or other evidence synthesis?	104 (100)	NA	0 (0)
13. Did the review authors account for RoB in individual studies when interpreting/discussing the results of the review?^a^	104 (100)	NA	0 (0)
14. Did the review authors provide a satisfactory explanation for, and discussion of, any heterogeneity observed in the results of the review?	104 (100)	NA	0 (0)
15. Did the review authors carry out an adequate investigation of publication bias (small study bias) and discuss its likely impact on the results of the review?^a^	104 (100)	NA	0 (0)
16. Did the review authors report any potential sources of conflict of interest, including any funding they received for conducting the review?	103 (99.0)	NA	1 (1.0)

Abbreviations: AMSTAR 2, A MeaSurement Tool to Assess systematic Reviews 2; NA, not applicable.

^a^ Critical domain items.

The performance of the following two domains was considered unsatisfactory, with less than 50% of MAs meeting relevant criteria: (i) 47 (45.2%) MAs provided an explicit statement that their methods were established before conducting the review and justified significant deviations from the protocol (Item 2); (ii) 29 (27.9%) MAs used a comprehensive literature search strategy (Item 4); and (iii) 7 (6.7%) MAs clearly listed excluded primary studies and justified study exclusions (Item 7). All of these are critical AMSTAR 2 items.

#### 3.3.2 Overall Methodological Quality

Table 3 illustrates the overall methodological quality of the included MAs by bibliographical characteristics. In general, the overall methodological quality of the 104 MAs was unsatisfactory, with only three (2.9%) judged to be of high quality. Over three‐quarters (82 MAs; 78.8%) were of critically low quality and 19 (18.3%) were of low quality. None of them were of moderate quality.

**TABLE 3:** Overall methodological quality of the 104 included meta‐analyses on sleep disorder treatments by bibliographical characteristics.

Bibliographical characteristics	High^a^	Moderate^a^	Low^a^	Critically low^a^	*p*
Total included MAs	3 (2.9)	0 (0)	19 (18.3)	82 (78.8)	
Cochrane review					0.018^b^
No	2 (1.9)	0 (0)	19 (18.5)	82 (79.6)	
Yes	1 (100)	0 (0)	0 (0)	0 (0)	
Update of a previous MA					0.606
No	3 (2.9)	0 (0)	19 (18.5)	81 (78.6)	
Yes	0 (0)	0 (0)	0 (0)	1 (100)	
Reported intervention harms					0.892
No	1 (1.6)	0 (0)	12 (19.4)	49 (79.0)	
Yes	2 (4.8)	0 (0)	7 (16.7)	33 (78.6)	
Searched non‐English databases					0.880
No	2 (3.9)	0 (0)	9 (17.7)	40 (78.4)	
Yes	1 (1.9)	0 (0)	10 (18.9)	42 (79.2)	
Included a PRISMA‐like flow diagram					0.463
No	0 (0)	0 (0)	0 (0)	2 (100)	
Yes	3 (2.9)	0 (0)	19 (18.3)	80 (76.9)	
Location of corresponding author					< 0.001^b^
Europe	2 (15.4)	0 (0)	5 (38.5)	6 (46.2)	
America	0 (0)	0 (0)	1 (6.7)	14 (93.3)	
Asia	1 (1.4)	0 (0)	9 (12.7)	61 (85.9)	
Oceania	0 (0)	0 (0)	4 (80.0)	1 (20.0)	
Funding location					< 0.001^b^
Europe	2 (50.0)	0 (0)	1 (25.0)	1 (25.0)	
America	0 (0)	0 (0)	1 (20.0)	4 (80.0)	
Asia	1 (2.4)	0 (0)	6 (12.3)	35 (83.3)	
Not reported	0 (0)	0 (0)	8 (16.0)	42 (84.0)	
Type of interventions					0.056
Non‐pharmacological	2 (3.0)	0 (0)	17 (25.4)	48 (71.6)	
Pharmacological	1 (3.2)	0 (0)	2 (6.5)	28 (90.3)	
Both types	0 (0)	0 (0)	0 (0)	6 (100)	
Reported year of coverage of literature search					0.606
No	0 (0)	0 (0)	0 (0)	1 (100)	
Yes	3 (2.9)	0 (0)	19 (18.5)	81 (78.6)	
Search terms reported for one or more electronic databases					0.101
No research term	0 (0)	0 (0)	0 (0)	0 (0)	
Topics/free text/keywords/MeSH	1 (1.9)	0 (0)	7 (13.0)	46 (85.2)	
Full Boolean	2 (4.0)	0 (0)	12 (24.0)	36 (72.0)	
Readers are referred elsewhere for full search strategy	0 (0)	0 (0)	0 (0)	0 (0)	
Language of included primary studies					0.489
English and other languages	1 (2.5)	0 (0)	10 (25.0)	29 (72.5)	
English only	0 (0)	0 (0)	2 (1.9)	10 (9.6)	
Not reported	2 (3.8)	0 (0)	7 (13.5)	43 (82.7)	
Tools for assessing quality of primary studies					0.716
Cochrane risk of bias	2 (2.8)	0 (0)	12 (16.7)	58 (80.6)	
Jadad Scale	0 (0)	0 (0)	1 (20.0)	4 (80.8)	
More than one tools	0 (0)	0 (0)	0 (0)	2 (100)	
Others	1 (4.0)	0 (0)	6 (24.0)	18 (72.0)	

Abbreviations: MeSH, Medical Subject Headings; PRISMA, Preferred Reporting Items for Systematic Reviews and Meta‐Analysis; SR, systematic review.

^a^ Values are *N* (% in subgroup).

^b^ *p* value of Kruskal–Wallis test < 0.05.

Kruskal–Wallis tests revealed statistically significant differences in overall methodological quality across three bibliographical characteristics. When compared to non‐Cochrane reviews, Cochrane reviews were less likely to be of critically low quality (0% vs. 79.6%; *p* = 0.018). MAs with a corresponding author from Europe (15.4%) were more likely to be of high quality, compared to those having a corresponding author from Asia (1.0%), America (0%), and Oceania (0%) (*p* < 0.001). Similarly, MAs that received funding from Europe (50.0%) tended to be of high quality, compared to those that received funding from Asia (2.4%), America (0%), and Oceania (0%) (*p* < 0.001). No bibliographical characteristics showed a statistically significant association with overall methodological quality based on Spearman's rank correlation coefficients.

## 4 Discussion

### 4.1 Summary of Results

We critically appraised 104 MAs on sleep disorder treatments published in the previous 5 years. They had an unsatisfactory overall methodological quality, with most (82 MAs; 78.9%) having critically low quality, 19 (18.3%) low quality, and only 3 (2.9%) high quality. In particular, they had poor performance in the AMSTAR 2 critical domains of (i) registering an a priori protocol to explain review methods and justifying any protocol deviations, (ii) using a comprehensive literature search strategy, and (iii) listing excluded primary studies and justifying study exclusions. We found statistically positive associations between overall methodological quality and (i) Cochrane reviews, (ii) MAs with the corresponding author from Europe, and (iii) MAs supported by funding from Europe.

### 4.2 Implications for Clinical Practice and Research

As many as three‐quarters of recently published MAs on the treatments of sleep disorders had an insufficient overall methodological quality. Such pitfalls in the study design, conduct, and reporting of MAs may cause underestimation or overestimation of treatment effectiveness, which may, in turn, mislead clinical and policy decision‐making based on biased findings (Schmucker et al. 2017). Therefore, healthcare professionals, policymakers, and other evidence users should consider conducting a critical appraisal of each MA before applying the evidence. Journal editors and peer reviewers should also be encouraged to follow updated methodological and reporting standards when assessing submissions.

Although only one Cochrane review is included in this review, the positive impact on MA methodological quality related to Cochrane reviews may somehow reflect the stringent requirements in review team composition and standardized review methodology in safeguarding the quality of the publications. It is not known whether the significant associations between MA methodological quality and European corresponding authors and fundings were merely due to the small sample size. That said, funding and resources should be allocated for supporting training in evidence synthesis and critical appraisal among researchers and clinicians. For future MA authors in this field, we propose the following recommendations to help improve the overall methodological quality.

### 4.3 Recommendations for Future MAs of Sleep Disorder Treatments

#### 4.3.1 Providing Lists of Excluded Studies With Rationale for Exclusion

Most (97 MAs; 93.3%) of the included MAs on sleep disorder treatments did not fulfill the criteria for providing a list of excluded studies and the justifications for their exclusion (Item 7). Failure to comply with relevant requirements is not uncommon, even in recent MAs on other interventions, such as acupuncture (Ho et al. 2021), atopic dermatitis (Ho et al. 2024), and sepsis (Ho et al. 2023). This phenomenon may be understandable because documenting excluded studies with reasons for exclusion is only compulsory for Cochrane reviews but not for MAs published in most journals (The Cochrane Collaboration 2020), so MA authors have no obligation or incentive to provide such information. However, this shortcoming may detract from the transparency and reproducibility of the MAs, given the inherent subjectivity in literature selection (Faggion 2015). Moreover, studies excluded by the authors due to restrictive inclusion criteria or exclusion errors might still be relevant and useful for addressing specific clinical questions (The Cochrane Collaboration 2020; Shea et al. 2017). We recommend that future MA authors provide a list of all potentially relevant primary studies that were read in full‐text form but excluded from the MA, along with sufficient justifications for their decision.

#### 4.3.2 Improving Comprehensiveness of Literature Search

Grey literature is produced at all levels of government, academia, business, and industry in both print and electronic formats but is not published by commercial publishers (Mahood, Van Eerd, and Irvin 2014). It has been estimated that grey literature accounts for one‐tenth of citations in nursing journals (Woods, Phillips, and Dudash 2020), and half of medical and health‐related studies are not published in peer‐reviewed journals (Song, Loke, and Hooper 2014). Despite the importance of grey literature in addressing gaps between peer‐reviewed publications, nearly three‐quarters (75 MAs; 72.1%) of the included MAs on sleep disorder treatments did not use comprehensive literature search strategies that cover unpublished documents and, therefore, failed to meet the criteria for this critical domain (Item 4). Similar weaknesses can be found in a majority of recent MAs in acupuncture (Ho et al. 2021), atopic dermatitis (Ho et al. 2024), and sepsis (Ho et al. 2023). This may be due to the fact that the literature search performed by MA authors tends to cover only major biomedical databases (e.g., MEDLINE, EMBASE, and the Cochrane Central Register of Controlled Trials), which is not considered comprehensive by the AMSTAR 2 (Shea et al. 2017). Therefore, considering the failure of searching unpublished documents, we are concerned about whether these 75 MAs are free from publication bias and capable of providing unbiased results applicable to clinical practice (McAuley et al. 2000). We recommend that future MA authors search various sources, including databases of public and private organizations, research‐related social media (e.g., LinkedIn and ResearchGate), and research‐related mailing lists (e.g., JISCMail) (Learning and Information Services UoW 2023).

#### 4.3.3 Registering a Priori Protocols and Justifying Protocol Deviation

Over half (*n* = 56; 53.9%) of MAs on sleep disorder treatments did not contain a registered protocol or an explicit statement that the review methods were established before the review (Item 2). This methodological flaw was also identified in recent MAs on the effectiveness of acupuncture (Ho et al. 2021), atopic dermatitis (Ho et al. 2024), and sepsis (Ho et al. 2023). On that account, we cannot guarantee the quality of the review process of those MAs by relying on previous peer reviews (for published protocols) or quality checks by administrators (for registered protocols) (Moher et al. 2015; The PLoS Medicine Editors 2011). We were also unable to identify whether the authors deviated from what they planned to do and whether they selectively reported positive findings (Moher et al. 2015; The PLoS Medicine Editors 2011). The transparency and replicability of the review process of the MAs are in doubt as well (Moher et al. 2015; The PLoS Medicine Editors 2011). Future reviewers should register their protocols on online platforms such as the International Prospective Register of Systematic Reviews (PROSPERO) (Centre for Reviews and Dissemination—University of York 2020), an international database of prospectively registered systematic reviews and MAs on health and social care, welfare, public health, education, crime, justice, and international development. Any deviations from protocols should be clearly reported and justified.

#### 4.3.4 Strengths and Limitations

This study has several strengths. First, we conducted critical appraisals of the MAs on sleep disorder treatments using the AMSTAR 2, a validated tool with adequate validity and inter‐rater reliability (Lorenz et al. 2019). Second, we conducted the literature search in three representative electronic databases (i.e., MEDLINE, EMBASE, and PsycINFO) for randomized controlled trials to ensure the relevance of our results. Third, this study investigated the overall methodological quality of the included MAs and emphasized their performance across each AMSTAR 2 domain. This approach offers valuable insights into potential areas of improvement and enables a comprehensive understanding of specific aspects that contributed to the low quality of MAs on sleep disorder treatments.

This study has several limitations. First, the critical appraisal results might be affected by the MAs' poor reporting quality, the publication journals' word limits, and the unavailability of online appendices of the MAs (The Cochrane Collaboration 2020; Shea et al. 2017). Second, we did not include studies published in languages other than English or unpublished documents, which might result in language bias and overlook relevant findings in grey literature. However, we believe that most MAs informing significant practice changes in this field are published in English and are peer‐reviewed, and our sample is likely to represent the MAs indexed in major international databases. Third, we excluded network MAs due to the lack of relevant critical appraisal tools. This exclusion may limit the completeness of the knowledge landscape of sleep disorder treatments. Fourth, we did not register the protocol for this review because the search and analytical approaches adopted were based on previous methodological reviews published in peer‐reviewed journals and were, therefore, standardized and validated.

## 5 Conclusions

The overall methodological quality of the MAs on sleep disorder treatments is unsatisfactory. This lack of quality may lead to unreliable conclusions about treatment effects and, consequently, jeopardize clinical and policy decision‐making. We recommend that future reviewers enhance the quality of their MAs by: (i) registering a priori protocols, (ii) using comprehensive literature search strategies, and (iii) providing a list of excluded studies and justifying these exclusions.

## Author Contributions

**Leonard Ho**: writing–original draft, methodology, formal analysis, data curation, conceptualization, visualization, project administration. **Yan Ling Kwok**: writing–original draft, data curation, validation, formal analysis. **Xi Chen**: writing–review and editing, validation. **Irene XY Wu**: writing–review and editing, methodology. **Chen Mao**: writing–review and editing, conceptualization. **Vincent Chi Ho Chung**: conceptualization, writing–review and editing, funding acquisition, supervision.

## Conflicts of Interest

The authors declare no conflicts of interest.

### Peer Review

The peer review history for this article is available at https://publons.com/publon/10.1002/brb3.70140.

## Supporting information



Table S1. Search strategies for meta‐analyses on treatments for sleep disorders
Table S2. 18‐Item bibliographical characteristics questionnaire
Table S3. AMSTAR 2 critical appraisal form and rating of overall methodological quality
Table S4. Excluded studies with reasons

## Data Availability

All data supporting the findings of this study are available within the paper and its Supporting Information.
