# Supplementary material for: A Methodological Quality Assessment of Meta‐Analyses on Sleep Disorder Treatments Using AMSTAR 2
Source: Brain Behav. 2024 Nov 17;14(11):e70140. doi: 10.1002/brb3.70140 (PMC11570419; doi:10.1002/brb3.70140)
Supplement: Supplementary file 1 — Table S1. Search strategies for meta‐analyses on treatments for sleep disorders Table S2. 18‐Item bibliographical characteristics questionnaire Table S3. AMSTAR 2 critical appraisal form and rating of overall methodological quality Table S4. Excluded studies with reasons [file BRB3-14-e70140-s001.docx]

**Methodological quality of meta-analyses on sleep disorder treatments: a cross-sectional study**

**Supplementary file 1**

**Table S1.** Search strategies for meta-analyses on treatments for sleep disorders

**Table S2.** 18-item bibliographical characteristics questionnaire

**Table S3.** AMSTAR 2 critical appraisal form and rating of overall methodological quality

**Table S4.** Excluded studies with reasons

**Table S1.** Search strategies for meta-analyses on treatments for sleep disorders

| **Ovid MEDLINE(R) 1946 to Current**   1. exp "SLEEP INITIATION AND MAINTENANCE DISORDERS"/ 2. insomni*.tw. 3. SLEEP/de 4. exp SLEEP STAGES/de 5. WAKEFULNESS/de 6. (sleep impact scale or sleep questionnaire or sleep scale or sleep evaluation questionnaire or sleep quality index or PSQI or sleep impairment index or sleepiness scale or sleep log or sleep diar*).tw. 7. (sleep adj3 (initiation or onset or maintenance)).tw. 8. (nocturnal adj (wake* or awake*)).tw. 9. 1 or 2 or 3 or 4 or 5 or 6 or 7 or 8 10. randomized controlled trial.pt. 11. randomized.mp. 12. placebo.mp. 13. 10 or 11 or 12 14. meta analysis.mp,pt. 15. review.pt. 16. search:.tw. 17. 14 or 15 or 16 18. 9 and 13 and 17 19. limit 18 to yr="2018-Current" |
| --- |
| **Embase 1910 to Current**   1. exp *SLEEP/ 2. exp *INSOMNIA/ 3. INSOMNIA/dt 4. (insomni* or sleep* or dyssomni* or wake* or awake* or chrono*).ti. 5. (sleep adj3 (initiation or onset or maintenance)).tw. 6. (nocturnal adj (wake* or awake*)).tw. 7. INSOMNIA SEVERITY INDEX/ 8. PITTSBURGH SLEEP QUALITY INDEX/ 9. EPWORTH SLEEPINESS SCALE/ 10. SLEEP PARAMETERS/ or SLEEP PATTERN/ or SLEEP QUALITY/ or SLEEP TIME/ 11. (insomnia rating scale* or WHIIRS or insomnia severity index or insomnia treatment scale or sleep impact scale or sleep questionnaire or sleep scale or sleep evaluation questionnaire or sleep quality index or PSQI or sleep impairment index or sleepiness scale or sleep log or sleep diar*).mp. 12. 1 or 2 or 3 or 4 or 5 or 6 or 7 or 8 or 9 or 10 or 11 13. random:.tw. 14. placebo:.mp. 15. double-blind:.tw. 16. 13 or 14 or 15 17. meta-analys:.mp. 18. search:.tw. 19. review.pt. 20. 17 or 18 or 19 21. 12 and 16 and 20 22. limit 21 to yr="2018-Current" |
| **APA PsycInfo 1806 to Current**   1. INSOMNIA/ 2. (insomni* or dyssomni*).ti,ab,id,tm. 3. (sleep impact or sleep questionnaire or sleep scale or sleep evaluation or sleep quality or PSQI or sleep impairment or sleepiness scale or sleep log or sleep diar*).ab,id,tm. 4. (sleep adj3 (initiation or onset or maintenance)).ti,ab,id. 5. (nocturnal adj (wake* or awake*)).ti,ab,id. 6. 1 or 2 or 3 or 4 or 5 7. double-blind.tw. 8. random: assigned.tw. 9. control.tw. 10. 7 or 8 or 9 11. control:.tw. 12. effectiveness.tw. 13. risk:.tw. 14. 11 or 12 or 13 15. 6 and 10 and 14 16. limit 15 to yr="2018-Current" |

**Table S2.** 18-item bibliographical characteristics questionnaire

| **Bibliographical Questions** | **Answers** |
| --- | --- |
| 1. Is there a Cochrane review? | Yes / No |
| 2. Is there an update of previous meta-analysis? | Yes / No |
| 3. Has harm been considered in the meta-analysis? | Yes / No |
| 4. Did the authors search for English databases? | Yes / No |
| 5. Did the authors search for non-English databases? | Yes / No |
| 6. Is there any PRISMA-like flow diagram in the review? | Yes / No |
| 7. Year of publication | ________ |
| 8. Impact factor of journal in the year before its publication | ________ |
| 9. Number of authors | ________ |
| 10. Number of all included studies | ________ |
| 11. Number of participants in all included studies | ________ |
| 12. Location of corresponding authors | 1. Europe 2. America 3. Asia 4. Oceania 5. Africa 6. Not reported |
| 13. Funding location of meta-analysis | 1. Europe 2. America 3. Asia 4. Oceania 5. Africa 6. Not reported |
| 14. Type of interventions | 1. Non-pharmacological 2. Pharmacological 3. Both types |
| 15. Was “year of coverage” reported? | 1. Yes (starting and ending years) 2. Partially (only starting years) 3. Not mentioned |
| 16. Searching terms reported | 1. No research terms 2. Topic/free text/keyword/MeSHs 3. Full Boolean 4. Readers are referred elsewhere for full search strategy |
| 17. Languages of the included primary studies in the meta-analysis | 1. English only 2. Language other than English 3. English and languages other than English 4. Language criteria not reported |
| 18. Tools for assessing quality of the primary studies | 1. Cochrane RoB 2. Jaded scale 3. Others |

MeSHs: Medical Subject Headings; PRISMA: Preferred Reporting Items for Systematic Reviews and Meta-Analyses; RCT: Randomised controlled trial; RoB: Risk of bias.

**Table S3.** AMSTAR 2 critical appraisal form and rating of overall methodological quality

| 1. **Did the research questions and inclusion criteria for the review include the components of PICO?**  \| For Yes  🞏 Population  🞏 Intervention  🞏 Comparator group  🞏 Outcome \| Optional (recommended)  🞏 Timeframe for follow-up \| **🞏 Yes**  **🞏 No** \| \| --- \| --- \| --- \| |
| --- | --- | --- | --- |
| 1. **Did the report of the review contain an explicit statement that the review methods were established prior to the conduct of the review and did the report justify any significant deviations from the protocol?**  \| For Partial Yes:  The authors state that they had a written protocol or guide that included ALL the following:  🞏 review question(s)  🞏 a search strategy  🞏 inclusion/exclusion criteria  🞏 a RoB assessment \| For Yes:  As for partial yes, plus the protocol should be registered and should also have specified:  🞏 a meta-analysis/synthesis  plan, if appropriate, and  🞏 a plan for investigating  causes of heterogeneity  🞏 justification for any  deviations from the protocol \| **🞏 Yes**  **🞏 Partial Yes**  **🞏 No** \| \| --- \| --- \| --- \| |
| 1. **Did the review authors explain their selection of the study designs for inclusion in the review?**  \| For Yes, the review should satisfy ONE of the following:  🞏 Explanation for including only RCTs  🞏 OR Explanation for including only NRSI  🞏 OR Explanation for including both RCTs and NRSI \| **🞏 Yes**  **🞏 No** \| \| --- \| --- \| |
| 1. **Did the review authors use a comprehensive literature search strategy?**  \| For Partial Yes (all the following):  🞏 searched at least 2 databases (relevant to research question)  🞏 provided key word and/or search strategy  🞏 justified publication restrictions (eg, language) \| For Yes, should also have (all the following):  🞏 searched the reference lists/bibliographies of included studies  🞏 searched trial/study registries  🞏 included/consulted content experts in the field  🞏 where relevant, searched for grey literature  🞏 conducted search within 24 months of completion of the review \| **🞏 Yes**  **🞏 Partial Yes**  **🞏 No** \| \| --- \| --- \| --- \| |
| 1. **Did the review authors perform study selection in duplicate?**  \| For Yes, either ONE of the following:  🞏 at least two reviewers independently agreed on selection of eligible studies and achieved consensus on which studies to include  🞏 OR two reviewers selected a sample of eligible studies and achieved good agreement (at least 80 per cent), with the remainder selected by one reviewer \| **🞏 Yes**  **🞏 No** \| \| --- \| --- \| |
| 1. **Did the review authors perform data extraction in duplicate?**  \| For Yes, either ONE of the following:  🞏 at least two reviewers achieved consensus on which data to extract from included studies  🞏 OR two reviewers extracted data from a sample of eligible studies and achieved good agreement (at least 80 per cent), with the remainder extracted by one reviewer \| **🞏 Yes**  **🞏 No** \| \| --- \| --- \| |
| 1. **Did the review authors provide a list of excluded studies and justify the exclusions?**  \| For Partial Yes:  🞏 provided a list of all potentially relevant studies that were read in full text form but excluded from the review \| For Yes, must also have:  🞏 Justified the exclusion from the review of each potentially relevant study \| **🞏 Yes**  **🞏 Partial Yes**  **🞏 No** \| \| --- \| --- \| --- \| |
| 1. **Did the review authors describe the included studies in adequate detail?**  \| For Partial Yes (ALL the following):  🞏 described populations  🞏 described interventions  🞏 described comparators  🞏 described outcomes  🞏 described research designs \| For Yes, should also have ALL the following:  🞏 described populations in detail  🞏 described intervention and comparator in detail (including does where relevant)  🞏 described study’s setting  🞏 timeframe for follow-up \| **🞏 Yes**  **🞏 Partial Yes**  **🞏 No** \| \| --- \| --- \| --- \| |
| 1. **Did the review authors use a satisfactory technique for assessing the RoB in individual studies that were included in the review?**  \| **RCTs**  For Partial Yes, must have assessed RoB from  🞏 unconcealed allocation, *and*  🞏 lack of blinding of patients and assessors when assessing outcomes (unnecessary for objective outcomes such as all cause mortality) \| For Yes, must also have assessed RoB from:  🞏 allocation sequence that was not truly random, *and*  🞏 selection of the reported result from among multiple measurements or analyses of a specified outcome \| **🞏 Yes**  **🞏 Partial Yes**  **🞏 No**  **🞏 Includes only NRSI** \| \| --- \| --- \| --- \| \| **NRSI**  For Partial Yes, must have assessed RoB:  🞏 from confounding, *and*  🞏 from selection bias \| For Yes, must also have assessed RoB:  🞏 methods used to ascertain exposures and outcomes, *and*  🞏 selection o the reported result from among multiple measurements or analyses of a specified outcome \| **🞏 Yes**  **🞏 Partial Yes**  **🞏 No**  **🞏 Includes only RCTs** \| |
| 1. **Did the review authors report on the sources of funding for the studies included in the review?**  \| For Yes  🞏 Must have reported on the sources of funding for individual studies included in the review. Note: Reporting that the reviewers looked for this information but it was not reported by study authors also qualifies \| **🞏 Yes**  **🞏 No** \| \| --- \| --- \| |
| 1. **If meta-analysis was performed did the review authors use appropriate methods for statistical combination of results?**  \| **RCTs**  For Yes:  🞏 The authors justified combining the data in a meta-analysis  🞏 AND they used an appropriate weighted technique to combine study results and adjusted for heterogeneity if present  🞏 AND investigated the causes of any heterogeneity \| **🞏 Yes**  **🞏 No**  **🞏 No meta-analysis conducted** \| \| --- \| --- \| \| **For NRSI**  For Yes  🞏 The authors justified combining the data in a meta-analysis  🞏 AND they used an appropriate weighted technique to combine study results, adjusting for heterogeneity if present  🞏 AND they statistically combined effect estimates from NRSI that were adjusted for confounding, rather than combining raw data, or justified combining raw data when adjusted effect estimates were not available  🞏 AND they reported separate summary estimates for RCTs and NRSI separately when both were included in the review \| **🞏 Yes**  **🞏 No**  **🞏 No meta-analysis conducted** \| |
| 1. **If meta-analysis was performed, did the review authors assess the potential impact of RoB in individual studies on the results of the meta-analysis or other evidence synthesis?**  \| For Yes  🞏 included only low RoB RCTs  🞏 OR, if the pooled estimate was based on RCTs and/or NRSI at variable RoB, the authors performed analyses to investigate possible impact of RoB on summary estimates of effect \| **🞏 Yes**  **🞏 No**  **🞎No meta-analysis conducted** \| \| --- \| --- \| |
| 1. **Did the review authors account for RoB in individual studies when interpreting/discussing the results of the review?**  \| For Yes:  🞏 included only low RoB RCTs  🞏 OR, if RCTs with moderate or high RoB, or NRSI were included the review provided a discussion of the likely impact of RoB on the results \| **🞏 Yes**  **🞏 No** \| \| --- \| --- \| |
| 1. **Did the review authors provide a satisfactory explanation for, and discussion of, any heterogeneity observed in the results of the review?**  \| For Yes:  🞏 There was no significant heterogeneity in the results  🞏 OR if heterogeneity was present the authors performed an investigation of sources of any heterogeneity in the results and discussed the impact of this on the results of the review \| **🞏 Yes**  **🞏 No** \| \| --- \| --- \| |
| 1. **If they performed quantitative synthesis did the review authors carry out an adequate investigation of publication bias (small study bias) and discuss its likely impact on the results of the review?**  \| For Yes:  🞏 performed graphical or statistical tests for publication bias and discussed the likelihood and magnitude of impact of publication bias \| **🞏 Yes**  **🞏 No**  **🞏 No meta-analysis conducted** \| \| --- \| --- \| |
| 1. **Did the review authors report any potential sources of conflict of interest, including any funding they received for conducting the review?**  \| For Yes:  🞏 The authors reported no competing interests OR  🞏 The authors described their funding sources and how they managed potential conflicts of interest \| **🞏 Yes**  **🞏 No** \| \| --- \| --- \|   **Rating of overall methodological quality**  **Critical items: 2, 4, 7, 9, 11, 13, 15**  **High**  No or one non-critical weakness: the systematic review provides an accurate and comprehensive summary of the results of the available studies that address the question of interest  **Moderate**  More than one non-critical weakness*: the systematic review has more than one weakness but no critical flaws. It may provide an accurate summary of the results of the available studies that were included in the review  **Low**  One critical flaw with or without non-critical weaknesses: the review has a critical flaw and may not provide an accurate and comprehensive summary of the available studies that address the question of interest  **Critically low**  More than one critical flaw with or without non-critical weaknesses: the review has more than one critical flaw and should not be relied on to provide an accurate and comprehensive summary of the available studies  *Multiple non-critical weaknesses may diminish confidence in the review and it may be appropriate to move the overall appraisal down from moderate to low confidence |

NRSI: Non-randomised studies of interventions; RCT: Randomised controlled trial; RoB: Risk of bias.

*Adapted from: Shea BJ, Reeves BC, Wells G, Thuku M, Hamel C, Moran J, et al. AMSTAR 2: a critical appraisal tool for systematic reviews that include randomised or non-randomised studies of healthcare interventions, or both. BMJ. 2017;358:j4008.*

**Table S4.** Excluded studies with reasons

| **Number** | **Full reference** | **Reasons** |
| --- | --- | --- |
| 1 | Abboud M. Vitamin D Supplementation and Sleep: A Systematic Review and Meta-Analysis of Intervention Studies. Nutrients. 2022 Mar 3;14(5):1076. | Not focus on sleep disorders |
| 2 | Al-Sadawi M, Saeidifard F, Kort S, Cao K, Capric V, Salciccioli L, Al-Ajam M, Budzikowski AS. Treatment of Sleep Apnea with Positive Airway Pressure and Its Association with Diastolic Dysfunction: A Systematic Review and Meta-Analysis. Respiration. 2022;101(3):334-344. | Focus on the treatment effects on complications or other symptoms |
| 3 | Alimoradi Z, Jafari E, Broström A, Ohayon MM, Lin CY, Griffiths MD, Blom K, Jernelöv S, Kaldo V, Pakpour AH. Effects of cognitive behavioral therapy for insomnia (CBT-I) on quality of life: A systematic review and meta-analysis. Sleep Med Rev. 2022 Aug;64:101646. | Focus on the treatment effects on complications or other symptoms |
| 4 | Ballesio A, Bacaro V, Vacca M, Chirico A, Lucidi F, Riemann D, Baglioni C, Lombardo C. Does cognitive behaviour therapy for insomnia reduce repetitive negative thinking and sleep-related worry beliefs? A systematic review and meta-analysis. Sleep Med Rev. 2021 Feb;55:101378. | Focus on the treatment effects on complications or other symptoms |
| 5 | Bandyopadhyay A, Kaneshiro K, Camacho M. Effect of myofunctional therapy on children with obstructive sleep apnea: a meta-analysis. Sleep Med. 2020;75:210-217. | Systematic review including observational studies |
| 6 | Bhagavan C, Kung S, Doppen M, et al. Cannabinoids in the Treatment of Insomnia Disorder: A Systematic Review and Meta-Analysis. CNS Drugs. 2020;34(12):1217-1228. | Systematic review including observational studies |
| 7 | Byun YJ, Yan F, Nguyen SA, Lentsch EJ. Transcutaneous Electrical Stimulation Therapy in Obstructive Sleep Apnea: A Systematic Review and Meta-analysis. Otolaryngol Head Neck Surg. 2020;163(4):645-653. | Systematic review including observational studies |
| 8 | Cattazzo F, Pengo MF, Giontella A, Soranna D, Bilo G, Zambon A, Karalliedde J, Gnudi L, Martinez-Garcia MÁ , Minuz P, Lombardi C, Parati G, Fava C. Effect of Continuous Positive Airway Pressure on Glucose and Lipid Profiles in Patients With Obstructive Sleep Apnoea: A Systematic Review and Meta-Analysis of Randomized Controlled Trials. Arch Bronconeumol. 2023 Mar 21:S0300-2896(23)00111-4. | Focus on the treatment effects on complications or other symptoms |
| 9 | Lehert P, Szoeke C. Comparison of modafinil and pitolisant in narcolepsy: a non-inferiority meta-analytical approach. Drugs Context. 2020;9:2020-6-2. | Network meta-analysis |
| 10 | Chalegre ST, Lins-Filho OL, Lustosa TC, França MV, Couto TLG, Drager LF, Lorenzi-Filho G, Bittencourt MS, Pedrosa RP. Impact of CPAP on arterial stiffness in patients with obstructive sleep apnea: a meta-analysis of randomized trials. Sleep Breath. 2021 Sep;25(3):1195-1202. | Focus on the treatment effects on complications or other symptoms |
| 11 | Chandler L, Patel C, Lovecka L, Gardani M, Walasek L, Ellis J, Meyer C, Johnson S, Tang NKY. Improving university students' mental health using multi-component and single-component sleep interventions: A systematic review and meta-analysis. Sleep Med. 2022 Dec;100:354-363. | Not focus on sleep disorders |
| 12 | Chen B, Drager LF, Peker Y, Vgontzas AN, Phillips CL, Hoyos CM, Salles GF, Guo M, Li Y. Effect of Continuous Positive Airway Pressure on Weight and Local Adiposity in Adults with Obstructive Sleep Apnea: A Meta-Analysis. AnnAm Thorac Soc. 2021 Oct;18(10):1717-1727. | Focus on the treatment effects on complications or other symptoms |
| 13 | Chen H, Eckert DJ, van der Stelt PF, et al. Phenotypes of responders to mandibular advancement device therapy in obstructive sleep apnea patients: A systematic review and meta-analysis. Sleep Med Rev. 2020;49:101229. | Systematic review including observational studies |
| 14 | Chen Q, Lin G, Chen Y, Wu C, Li C, Huang J, Lin Q. Impact of CPAP treatment for obstructive sleep apnea on visceral adipose tissue: a meta analysis of randomized controlled trials. Sleep Breath. 2021 Jun;25(2):555-562. | Focus on the treatment effects on complications or other symptoms |
| 15 | Chen R, Huang ZW, Lin XF, Lin JF, Yang MJ. Effect of continuous positive airway pressure on albuminuria in patients with obstructive sleep apnea: a meta-analysis. Sleep Breath. 2022 Mar;26(1):279-285. | Focus on the treatment effects on complications or other symptoms |
| 16 | Cheung DST, Takemura N, Smith R, Yeung WF, Xu X, Ng AYM, Lee SF, LinCC. Effect of qigong for sleep disturbance-related symptom clusters in cancer: a systematic review and meta-analysis. Sleep Med. 2021 Sep;85:108-122. | Not focus on sleep disorders |
| 17 | Dai Y, Liu J. Omega-3 long-chain polyunsaturated fatty acid and sleep: a systematic review and meta-analysis of randomized controlled trials and longitudinal studies. Nutr Rev. 2021 Jul 7;79(8):847-868. | Not focus on sleep disorders |
| 18 | Dontsos VK, Chatzigianni A, Papadopoulos MA, Nena E, Steiropoulos P. Upper airway volumetric changes of obstructive sleep apnoea patients treated with oral appliances: a systematic review and meta-analysis. Eur J Orthod. 2021;43(4):399-407. | Systematic review including observational studies |
| 19 | Du F, Gu YH, He YC, Deng WF, Liu ZZ. High-flow nasal cannula therapy for pediatric obstructive sleep apnea: a systematic review and meta-analysis. Eur Rev Med Pharmacol Sci. 2022;26(13):4583-4591. | Systematic review including observational studies |
| 20 | Duan J, Xia W, Yang K, et al. The Efficacy of Twin-Block Appliances for the Treatment of Obstructive Sleep Apnea in Children: A Systematic Review and Meta-Analysis. Biomed Res Int. 2022;2022:3594162. | Systematic review including observational studies |
| 21 | Fletcher HV, Cho PSP, Loong SL, Estrada-Petrocelli L, Patel AS, Birring SS,Lee KK. Effect of continuous positive airway pressure on maximal exercise capacity in patients with obstructive sleep apnea: a systematic review and meta-analysis. J Clin Sleep Med. 2020 Nov 15;16(11):1847-1855. | Focus on the treatment effects on complications or other symptoms |
| 22 | Green M, Ken-Dror G, Fluck D, Sada C, Sharma P, Fry CH, Han TS. Meta analysis of changes in the levels of catecholamines and blood pressure with continuous positive airway pressure therapy in obstructive sleep apnea. J ClinHypertens (Greenwich). 2021 Jan;23(1):12-20. | Focus on the treatment effects on complications or other symptoms |
| 23 | Herrera Y, Poon J, Ho KS, Pattupara A, Steiger D, Jean R. Cardiovascular outcomes in patients with sleep apnea treated with positive airway pressure: meta-analysis of randomized controlled trials. Chest. 2020;158(4):A2350. | Focus on the treatment effects on complications or other symptoms |
| 24 | Howell M, Avidan AY, Foldvary-Schaefer N, et al. Management of REM sleep behavior disorder: an American Academy of Sleep Medicine systematic review, meta-analysis, and GRADE assessment. J Clin Sleep Med. 2023;19(4):769-810. | Systematic review including observational studies |
| 25 | Hu Y, Su Y, Hu S, Ma J, Zhang Z, Fang F, Guan J. Effects of telemedicine interventions in improving continuous positive airway pressure adherence inpatients with obstructive sleep apnoea: a meta-analysis of randomised controlled trials. Sleep Breath. 2021 Dec;25(4):1761-1771. | Not focus on treatments for sleep disorders |
| 26 | Kang KT, Yeh TH, Hsu YS, et al. Effect of Sleep Surgery on C-Reactive Protein Levels in Adults With Obstructive Sleep Apnea: A Meta-Analysis. Laryngoscope. 2021;131(5):1180-1187. | Systematic review including observational studies |
| 27 | Ken-Dror G, Fry CH, Murray P, Fluck D, Han TS. Changes in cortisol levels by continuous positive airway pressure in patients with obstructive sleep apnoea: Meta-analysis of 637 individuals. Clin Endocrinol (Oxf). 2021 Dec;95(6):909-917. | Focus on the treatment effects on complications or other symptoms |
| 28 | Kwak KH, Lee YJ, Lee JY, Cho JH, Choi JH. The Effect of Pharyngeal Surgery on Positive Airway Pressure Therapy in Obstructive Sleep Apnea: A Meta-Analysis. J Clin Med. 2022;11(21):6443. | Systematic review including observational studies |
| 29 | Labarca G, Schmidt A, Dreyse J, Jorquera J, Enos D, Torres G, Barbe F. Efficacy of continuous positive airway pressure (CPAP) in patients with obstructive sleep apnea (OSA) and resistant hypertension (RH): Systematic review and meta-analysis. Sleep Med Rev. 2021 Aug;58:101446. | Focus on the treatment effects on complications or other symptoms |
| 30 | Lechien JR, Chiesa-Estomba CM, Fakhry N, et al. Surgical, clinical, and functional outcomes of transoral robotic surgery used in sleep surgery for obstructive sleep apnea syndrome: A systematic review and meta-analysis. Head Neck. 2021;43(7):2216-2239. | Systematic review including observational studies |
| 31 | Lee CH, Hsu WC, Yeh TH, Ko JY, Lin MT, Kang KT. Effect of Sleep Surgery on Inflammatory Cytokines in Adult Obstructive Sleep Apnea: A Systematic Review and Meta-Analysis. Laryngoscope. 2022;132(11):2275-2284. | Systematic review including observational studies |
| 32 | Lee CH, Hsu WC, Yeh TH, Ko JY, Lin MT, Kang KT. Effect of sleep surgery on lipid profiles in adults with obstructive sleep apnea: a meta-analysis. Eur Arch Otorhinolaryngol. 2022;279(8):3811-3820. | Systematic review including observational studies |
| 33 | Li J, Yan W, Yi M, Lin R, Huang Z, Zhang Y. Efficacy of CPAP duration and adherence for cognitive improvement in patients with obstructive sleep apnea: a meta-analysis of randomized controlled trials. Sleep Breath. 2022 Aug 5. | Focus on the treatment effects on complications or other symptoms |
| 34 | Li X, Zhou X, Xu X, Dai J, Chen C, Ma L, Li J, Mao W, Zhu M. Effects of continuous positive airway pressure treatment in obstructive sleep apnea patients with atrial fibrillation: A meta-analysis. Medicine (Baltimore). 2021 Apr 16;100(15):e25438. | Systematic review including observational studies |
| 35 | Lin CY, Chiang CH, Tseng MM, Tam KW, Loh EW. Effects of quetiapine on sleep: A systematic review and meta-analysis of clinical trials. Eur Neuropsychopharmacol. 2023 Feb;67:22-36. | Not focus on sleep disorders |
| 36 | Lin HJ, Yeh JH, Hsieh MT, Hsu CY. Continuous positive airway pressure with good adherence can reduce risk of stroke in patients with moderate to severe obstructive sleep apnea: An updated systematic review and meta-analysis. Sleep Med Rev. 2020;54:101354. | Systematic review including observational studies |
| 37 | Lin J, Hu S, Shi Y, Lu F, Luo W, Lin Y. Effects of continuous positive airway pressure on plasma fibrinogen levels in obstructive sleep apnea patients: a systemic review and meta-analysis. Biosci Rep. 2021 Jan 29;41(1):BSR20203856. | Focus on the treatment effects on complications or other symptoms |
| 38 | Lisan Q, Baudouin R, Lechien JR, Hans S, Blumen M. Is drug-induced sleep endoscopy associated with better outcomes after soft tissue surgery for sleep apnea? A systematic review and meta-analysis. Clin Otolaryngol. 2023;48(2):122-129. | Systematic review including observational studies |
| 39 | Liu Y, Li C, Wu C, Li P, Su Y, Chen Q. Efficacy of continuous positive airway pressure on subcutaneous adipose tissue in patients with obstructive sleep apnea: a meta-analysis of randomized controlled trials. Sleep Breath. 2021 Mar;25(1):1-8. | Focus on the treatment effects on complications or other symptoms |
| 40 | Mecenas P, Miranda GHN, Fagundes NCF, Normando D, Ribeiro KCF. Effects of oral appliances on serum cytokines in adults with obstructive sleep apnea: a systematic review. Sleep Breath. 2022 Sep;26(3):1447-1458. | Focus on the treatment effects on complications or other symptoms |
| 41 | Park J, Render PharmD KP, Cates PharmD DW. Daridorexant: Comprehensive Review of A New Oral Agent for the Treatment of Insomnia. Ann Pharmacother. 2023 Jan 5:10600280221143794. | No meta-analysis |
| 42 | Pengo MF, Soranna D, Giontella A, Perger E, Mattaliano P, Schwarz EI,Lombardi C, Bilo G, Zambon A, Steier J, Parati G, Minuz P, Fava C. Obstructive sleep apnoea treatment and blood pressure: which phenotypes predict a response? A systematic review and meta-analysis. Eur Respir J.2020 May 7;55(5):1901945. | Focus on the treatment effects on complications or other symptoms |
| 43 | Ruan J, Chen S, Liang J, Mak YW, Yee Ho FY, Chung KF, Kwun Tong AK, Zhang XL, Yeung WF. Acceptance and commitment therapy for insomnia and sleep quality: A systematic review and meta-analysis. J Contextual Behav Sci. 2022;26:139-155. | Systematic review including observational studies |
| 44 | Saenwandee P, Neruntarat C, Saengthong P, et al. Barbed pharyngoplasty for obstructive sleep apnea: A meta-analysis. Am J Otolaryngol. 2022;43(2):103306. | Systematic review including observational studies |
| 45 | Shang W, Zhang Y, Wang G, Han D. Benefits of continuous positive airway pressure on glycaemic control and insulin resistance in patients with type 2 diabetes and obstructive sleep apnoea: A meta-analysis. Diabetes Obes Metab. 2021;23(2):540-548. | Focus on the treatment effects on complications or other symptoms |
| 46 | Shinjyo N, Waddell G, Green J. Valerian Root in Treating Sleep Problems and Associated Disorders-A Systematic Review and Meta-Analysis. J Evid Based Integr Med. 2020;25:2515690X20967323. | Systematic review including observational studies |
| 47 | Staines AC, Broomfield N, Pass L, Orchard F, Bridges J. Do non pharmacological sleep interventions affect anxiety symptoms? A meta analysis. J Sleep Res. 2022 Feb;31(1):e13451. | Focus on the treatment effects on complications or other symptoms |
| 48 | Sutanto CN, Loh WW, Kim JE. The impact of tryptophan supplementation on sleep quality: a systematic review, meta-analysis, and meta-regression. Nutr Rev. 2022 Jan 10;80(2):306-316. | Not focus on sleep disorders |
| 49 | Tan JSI, Cheng LJ, Chan EY, Lau Y, Lau ST. Light therapy for sleep disturbances in older adults with dementia: a systematic review, meta-analysis and meta-regression. Sleep Med. 2022 Feb;90:153-166. | Not focus on sleep disorders |
| 50 | Thakral M, Von Korff M, McCurry SM, Morin CM, Vitiello MV. Changes in dysfunctional beliefs about sleep after cognitive behavioral therapy for insomnia: A systematic literature review and meta-analysis. Sleep Med Rev.2020 Feb;49:101230. | Focus on the treatment effects on complications or other symptoms |
| 51 | Tian Z, Xiao J, Kang J, Sun H, Mu Z, Tong D, Li M. Effects of Continuous Positive Airway Pressure on Cell Adhesion Molecules in Patients with Obstructive Sleep Apnea: A Meta-Analysis. Lung. 2021 Dec;199(6):639-651. | Focus on the treatment effects on complications or other symptoms |
| 52 | Timkova V, Nagyova I, Reijneveld SA, Tkacova R, van Dijk JP, Bültmann U. Quality of life of obstructive sleep apnoea patients receiving continuous positive airway pressure treatment: A systematic review and meta-analysis. Heart Lung. 2020 Jan-Feb;49(1):10-24. | Focus on the treatment effects on complications or other symptoms |
| 53 | Tsolakis IA, Palomo JM, Matthaios S, Tsolakis AI. Dental and Skeletal Side Effects of Oral Appliances Used for the Treatment of Obstructive Sleep Apnea and Snoring in Adult Patients-A Systematic Review and Meta-Analysis. J Pers Med. 2022 Mar 16;12(3):483. | Systematic review including observational studies |
| 54 | Wang J, Covassin N, Dai T, et al. Therapeutic value of treating central sleep apnea by adaptive servo-ventilation in patients with heart failure: A systematic review and meta-analysis. Heart Lung. 2021;50(2):344-351. | Systematic review including observational studies |
| 55 | Zhou N, Ho JTF, Huang Z, et al. Maxillomandibular advancement versus multilevel surgery for treatment of obstructive sleep apnea: A systematic review and meta-analysis. Sleep Med Rev. 2021;57:101471. | Systematic review including observational studies |
